# Diseases of the Lungs and Pleuræ

**Published:** 1895-12-28

**Authors:** 


### Progress in Medicine.

### DISEASES OF THE LUNGS AND PLEURÆ.

**Tuberculosis.**—The importance of the early recognition of pulmonary tuberculosis is recognised by all practitioners. Yet many of the earlier manifestations are most obscure. Hanot[1] draws attention to some facts which it is well to bear in mind, when he remarks that sometimes the infecting micro-organism attacks the system as a whole; at other times, concealed in a minute fold of the skin, or of the mucosa lining the respiratory passages from the nose to the lungs, or the digestive tract from the mouth to the anus, or of the mucous membrane of the eye or ear, it awaits, as it were, a favourable opportunity for displaying its activity. Such an opportunity is afforded when the organism is impaired in some way so as to open, even without obvious solution of continuity: (1) the lymphatics, enabling the microbe to reach the venous circulation directly, or to pass, after a sojourn in the glands, through the large lymph collectors and thoracic duct into the venous system; (2) the veins, by which it is carried through the cardio-vascular paths to the arterial system; or (3) the arteries themselves, leading to general infection. But though after-birth infection may take place from almost every conceivable source, it may also occur in intra-uterine life, the assumption being that the morbid germ is deposited in the ovule before conception, or at the same time as the fecundating germ, but the fœtus also may be infected through the placental circulation, indeed, Baumgarten goes so far as to express the opinion that in almost all cases, even in adults, this disease is of fœtal origin. Hanot has long held that the morbific causes of disease which we are able to distinguish and analyse add their effect to the transformations already produced *ab ovo*, and, moreover, from the very fact that they are secondary, they undergo attenuation or aggravation, as determined by the previous state. He summarises his views on these points in the following words, "the pathogenic agent of tuberculosis is derived from every conceivable source, and enters the organism at any point on the surface, and at any period of life."

Hanot's statements on the relationship between catarrhal affections of the respiratory tract and phthisis are worthy of special note. He says that subjects who on various occasions have suffered from bronchitis, laryngitis, or even acute rhinitis, not infrequently ultimately become phthisical, and these recurrent inflammations may reasonably be assumed to represent attenuated and abortive attacks of tuberculosis, as has been proved by the frequent discovery of the tubercle bacillus in the sputum. In certain cases of acute tubercular bronchitis preceding pulmonary phthisis, a diagnosis may now be arrived at by examination of the sputum. Examination by auscultation of the condition of the parenchyma is sometimes negative, because the signs are so slight as to pass undetected by the average medical man, and also because it is masked by the presence of sibilant, sonorous râles, and by signs of vicarious emphysema. In some cases, however, the localisation—or, at any rate, the predominance at one of the apices—of the dry râles of acute bronchitis points the way to the true diagnosis. He has several times had under observation persons suffering from acute bronchitis, and presenting sibilant, sonorous râles disseminated in both lungs, but distinctly predominating over one apex, who became tuberculous within a longer or shorter time, this tuberculosis developing first at the apex, where the bronchitis predominated.

The relation between serous pleurisy and tuberculosis is discussed by Eichhorst,[2] of Zurich. He reminds us that Buss has found that only 33 per cent. of persons who have suffered from serous pleurisy were living at the end of five years. He injected 15 minims of serous fluid from serous pleurisies into the peritoneal cavities of eleven guinea-pigs, with the result that only one became tuberculous. But when he subsequently used larger injections, viz., ʒss., 65 per cent. of the guinea-pigs (15 out of 23) became tuberculous, and later on five of the 15 patients returned with tuberculosis. He concluded, therefore, that two-thirds of the cases of serous pleurisy are, in fact, tuberculous, and that, as a rule, recoveries in most cases are but conditional. The infection originates but rarely in the lungs, generally in the tracheobronchial glands.

**Treatment.**—Nucleins forms the title of an interesting paper by W. H. Porter.[3] Firstly, in answer to the question as to what nuclein is, we find it described as a tetra-basic acid, composed of carbon, hydrogen, nitrogen, phosphorus, and oxygen, with the formula $C_{29} H_{49} N_9 P_3 O_{22}$. The antecedent substance from which nuclein appears to be derived is a complex molecule, apparently the product of a synthetic process in plant life by which a few atoms of phosphorus and iron are made to enter into combination with the proteid molecule, and this more complex molecule has received the name of nucleo-albumin. Nucleo-albumin must be looked upon as a common constituent of all cell elements. It is quite probable that the original nucleo-albumin is a vegetable globulin to which phosphorus and iron atoms have been added.

Viewed in this light, he continues, nucleo-albumin is a synthetic product of vegetable chemistry, where anabolic construction is continually taking place. When this nucleo-albumin is oxidised, or split up, it yields the more simple molecule to which the name "nuclein" has been given. Nuclein was first made from pus, then from spermatozoa, blood corpuscles, etc. Wherever nuclei are present, a substance can be obtained from their nucleo-albumin, rich in phosphorus, soluble in weak alkalies, insoluble in acids and in artificial gastric juice, sticky in character somewhat like mucin, but differing from the latter in containing phosphorus. From this method of development it appears quite clear that the opinion held by many observers that nuclein is not a normal constituent of the animal economy is the correct one, but it is obtained from the splitting up of nucleo-albumin just as urea and uric acid are katabolic products resulting from the oxidation of the proteid molecule. Yet nuclein is itself an artificial product of the laboratory, and not found in the excretions. The curtailed phosphorus of the nucleo-albumin probably furnishes the phosphorus of lecithin, and phosphoric acid.

Porter argues that it will take its place as a valuable therapeutic agent if it can be shown that the therapeutic effects of nuclein are due to its stimulating the animal economy into more rapid chemico-physiological activity, so that the system can the more rapidly utilize the normal food-stuffs to develop a higher nutritive state, thus placing the system in a higher state of resistance against toxic agents of all kinds. So far, however, such evidence is not to be found.

Rosenberry[4] has tried the effect of nuclein clinically, and in recording his results he remarks that with a cell acting up to its highest possible efficiency, the multiplication and growth of pathogenic germs becomes impossible, and there can be no doubt but that the treatment of the future will consist in bringing the vital activity of the cell up to a normal standard. If the theory of phagocytosis of Metchinkoff be true, that there is a constant struggle between the white blood-cells and the various pathogenic germs which invade our bodies, it emphasises the statement that the great object to be obtained by the therapeutist is to confer immunity, to furnish all the cells of the body with abundant supply of appropriate pabulum, and to increase the number of white cells. That we have in nuclein a substance which furnishes this cell-food in a form in which it can be readily utilised seems quite certain. That it increases the number of white blood cells enormously has been demonstrated by the work of Vaughan, McClintock, and Warthin. Having had experimental knowledge of the action of nuclein in his own case, he takes this opportunity of recording it. Rosenberry, in February, 1894, was suffering from tuberculous kidneys, and tubercle bacilli were found in the pus in the urine. He went under the nuclein treatment at the hands of Dr. Vaughan. In March, half a drachm was given to begin with, and the quantity rapidly increased till the daily quantity injected reached 1½ drachms, when chills, fever, vomiting, and general malaise supervened. They were discontinued for a time, and the 1½ drachm doses then resumed. No pus ever formed at the site of injection, but with the larger doses there was a painful hot swelling for some days. His general health steadily improved; the local symptoms, which at no time amounted to more than slight uneasiness and a dull, heavy feeling at the neck of the bladder, disappeared, and at the time of his report the health was excellent.

Rosenberry has used the substance in two cases of pulmonary tuberculosis. The first was far advanced, a cavity having formed, there being hectic, night sweats, emaciation, &c. No effect was produced. In the second case the patient was treated for three weeks only, and, with a subsequent trip to Florida, the result appeared to be "a cure." Rosenberry believes that in nuclein we have a remedy of great value, especially in incipient stages of tubercular disease. It is absolutely innocuous, and is abundant and cheap. Where properly prepared and kept in glass-stoppered bottles it will keep indefinitely, but in corked bottles it decomposes and forms toxins.

Renzi[5] has given nuclein in parenchymatous injections, beginning with half a cubic centimetre and a quarter of a cubic centimetre daily, up to large doses of three and a quarter centimetres. These injections caused neither local nor general troubles. In most cases they were well borne. Of five patients treated, three showed marked general improvement; one showed considerable decreases of râles, but no great improvement.

Wordin[6] gives Vaughan's conclusions, cases being treated in their earliest stages only, and confirmed by presence of bacilli: (1) In pulmonary tuberculosis, which has progressed to the formation of cavities, nucleinic acid from yeast will not effect a cure. (2) Even when the tuberculosis is of long standing, and when the extent of tissue involved is great, so long as secondary infection with pyogenic germs has not occurred, the proper use of the remedy may retard (he does not say arrest) the progress of the disease. (3) In initial cases of pulmonary tuberculosis, when there is no secondary infection, and when the area involved is small, and the resistance of the patient is not too much reduced, the proper employment of this agent may produce at least a temporary cure. He uses the guarded expression "temporary cure" because none of his cases have been under observation a sufficient length of time for him to say that the bacilli will not reappear. (4) In the few cases of urinary tuberculosis that he has reported, the results have been remarkably satisfactory.

[1] Med. Week, Oct. 11, 1895. [2] New York Med. Rec., Nov. 9, 1895; Correspondenz-Blatt fur Schweizer Aerzte. [3] Yale Med. Journ., Nov. 1895. [4] Ther. Gaz., Nov. 15, 1895. [5] Intern. Med. Mag., Oct., 1895, Ther. Woch., VIII. 25, 1895. [6] Yale Med. Journ., Nov., 1895.

